# Huntington disease update: new insights into the role of repeat instability in disease pathogenesis

**DOI:** 10.1515/medgen-2021-2101

**Published:** 2022-01-12

**Authors:** Larissa Arning, Huu Phuc Nguyen

**Affiliations:** Department of Human Genetics, Medical Faculty, Ruhr-University Bochum, Bochum 44780, Germany

**Keywords:** Huntington disease, repeat expansion disorders, somatic repeat expansion, genetic modifiers

## Abstract

The causative mutation for Huntington disease (HD), an expanded trinucleotide repeat sequence in the first exon of the huntingtin gene (*HTT*) is naturally polymorphic and inevitably associated with disease symptoms above 39 CAG repeats. Although symptomatic medical therapies for HD can improve the motor and non-motor symptoms for affected patients, these drugs do not stop the ongoing neurodegeneration and progression of the disease, which results in severe motor and cognitive disability and death. To date, there is still an urgent need for the development of effective disease‐modifying therapies to slow or even stop the progression of HD. The increasing ability to intervene directly at the roots of the disease, namely *HTT* transcription and translation of its mRNA, makes it necessary to understand the pathogenesis of HD as precisely as possible.

In addition to the long-postulated toxicity of the polyglutamine-expanded mutant HTT protein, there is increasing evidence that the CAG repeat-containing RNA might also be directly involved in toxicity. Recent studies have identified *cis*- (DNA repair genes) and *trans*- (loss/duplication of CAA interruption) acting variants as major modifiers of age at onset (AO) and disease progression. More and more extensive data indicate that somatic instability functions as a driver for AO as well as disease progression and severity, not only in HD but also in other polyglutamine diseases. Thus, somatic expansions of repetitive DNA sequences may be essential to promote respective repeat lengths to reach a threshold leading to the overt neurodegenerative symptoms of trinucleotide diseases. These findings support somatic expansion as a potential therapeutic target in HD and related repeat expansion disorders.

## Introduction

Huntington disease (HD) is a slowly progressive and ultimately fatal neurodegenerative disorder, which is inherited in an autosomal dominant manner and characterized by movement disorders and changes in behavior and mental state. Typically, the motor defects include chorea and loss of coordination. Neuropsychiatric symptoms cover the entire spectrum of psychiatric illnesses, with depression, psychosis and obsessive-compulsive disorder most frequently occurring in the course of the illness and being particularly stressful for patients, relatives and caregivers [1].

In 1993, the DNA sequence and the precise nature of the HD-associated mutation in the *HTT* gene on chromosome 4 was determined. The underlying mutation is the expansion of a physiologically polymorphic CAG trinucleotide repeat in exon 1, which is translated into an elongated polyglutamine tract in the huntingtin protein (HTT) [2].

HD is the most common disorder of at least nine CAG/polyglutamine diseases, including several spinocerebellar ataxias (SCAs), in which CAG repeat expansions encode elongated stretches of glutamines in the respective entirely unrelated disease-associated proteins [3]. HD occurs worldwide but its occurrence varies widely, with the highest prevalence rates for HD reported for western populations from Europe with up to 12 per 100,000 [4].

HTT is a soluble largely α-helical 3,144-amino-acid (348-kDa) protein, essential for embryonic development and involved in cellular activities such as vesicular transport and recycling, endocytosis, endosomal trafficking, autophagy and transcription regulation; however, the entire range of its normal function(s) still remains incompletely defined [5].

The average CAG repeat length in the general population comprises 16–20 repeats; 36 or more CAG units are pathogenic, with repeat lengths of 36–39 CAGs considered reduced-penetrance (RP) alleles [6]. Individuals who carry an expanded *HTT* allele can become symptomatic at any time point in their life and be healthy until then with no apparent signs of the disease. In the vast majority of cases, the clinical course of HD slowly begins in adulthood, typically in the mid-40s; the age of onset (AO) of disease refers to the time where pre-manifest mutation carriers convert into symptomatic HD patients. Formally, this point of time is reached when the first characteristic motor signs like chorea, bradykinesia or dystonia become overt [7].

The dominant genetic transmission and the fact that AO, phenotype and disease progression do not significantly differ between homozygotes and heterozygotes early led to the proposal of a toxic gain-of-function mechanism that acts through augmentation or dysregulation of one or more normal functions of HTT, either at the RNA or the protein level.

## RNA-dependent mechanisms in the molecular pathogenesis of HD

Although aggregated protein fragments are the histopathological hallmark of not only HD but also several other neurodegenerative diseases, their exact role remains controversial. From early on, a predominant hypothesis of toxicity in HD was protein misfolding and accumulation of insoluble aggregates that trigger neuronal dysfunction and lead to cell death [8], [9], [10], [11]. Therefore, prevention of aggregate formation appeared to be a universal strategy to reduce toxicity in HD and other polyglutamine diseases. Since the observation that N-terminal fragments of HTT accumulate in the nucleus, cytoplasm, dendrites and neurites of neurons in HD affected brains, many studies have supported the idea that the generation of small fragments plays a critical role in HD pathogenesis, whereas the formation of large aggregates might be part of a protective cellular mechanism to sequester soluble toxic species [10], [12]. Initially, the proteolytic cleavage of mutant HTT by caspases, calpains and other endoproteases was identified to be involved in the formation of N-terminal fragments [13], [14]. In the meantime, however, it has also been shown that aberrant splicing events at the RNA level play a role in the formation of these fragments. Beside the full-length *HTT* transcripts that differ in their 3′ UTR lengths, also CAG repeat length-dependent aberrant splicing of exon 1 resulting in short polyadenylated mRNAs that are translated into highly pathogenic exon 1 HTT proteins, both in human and mouse, has been described [15], [16].

Furthermore, it has previously been shown that CAG and CUG expansion transcripts can undergo a novel type of protein translation in which homopolymeric proteins are expressed in all three reading frames without an ATG initiation codon. These repeat-associated non-AUG (RAN) translated products are abundantly expressed in affected regions of HD autopsy brains [17]. However, whether RAN-translated products contribute to disease pathogenesis remains unknown [18]. But it is becoming apparent that beside the abnormal function of the mutant protein, also direct detrimental effects of RNA or RNA-dependent mechanisms could provide a mechanistic basis for the molecular pathogenesis of HD and should be considered in the development of RNA-targeted therapies to lower HTT levels [19].

## Genetic modifiers – DNA repair pathways, especially mismatch repair, play a central role in the pathogenesis of HD

As soon as the number of CAG triplets exceeds 40, the length of the CAG repeat in the expanded *HTT* disease allele inversely correlates with the AO and explains up to 70 % of its variability. Especially in patients with the typical CAG repeat range (40–55 CAG triplets), which is associated with mid-life adult onset of disease, considerable variation of up to 40 years in AO of neurological symptoms is described, even among individuals with identical repeat lengths. This onset variability in patients bearing the same mutations emphasizes the role of functional genetic differences in the genome of these patients that could modify the rate and onset of pathogenesis.

Genetic research approaches in HD can therefore help to understand the differences in clinical onset observed between patients, as well as to identify novel biomarkers and therapeutic strategies to improve disease management. Various human genetic strategies have been used in the last two decades in order to search for these disease-modifying factors that act before clinical diagnosis [20].

Until some years ago, the effort of discovering genetic modifiers was dominated by targeted approaches in which specific genes and variants were chosen on the basis of known or suspected participation in the disease pathology or to functional interaction with the (elongated) HTT protein. A number of *trans* modifiers in different genes have since been proposed as genetic HD modifiers, which could, however, not yet be replicated in the newer genome-wide association studies (GWAS) applying the stringent genome-wide significance threshold [20].

Interestingly, the GWAS in large patient cohorts all provide evidence for variation in DNA repair genes that modify the AO [21], [22], [23]. The first GWAS of 4,082 HD patients using the difference between AO predicted by CAG length and actual AO of motor symptoms identified *FAN1* (FANCD2 and FANCI associated nuclease 1) on chromosome 15 and *RRM2B* (ribonucleotide reductase regulatory TP53 inducible subunit M2B) on chromosome 8 [21]. In addition, a suggestive association signal was detected on chromosome 3p22.2 near *MLH1* (mutL homolog 1), a tumor suppressor gene involved in DNA mismatch repair.

Further candidate single nucleotide polymorphism (SNP) analyses in a cohort of 3,314 additional HD patients independently confirmed the chromosome 8 and 15 loci and moved the *MLH1* association to genome-wide significance [24]. Interestingly, the ortholog of *MLH1* has previously been indicated as a potential genetic modifier of strain-specific HTT CAG instability [25]. Furthermore, MSH3 (MutS homolog 3), another member of the DNA mismatch repair proteins that has been extensively implicated in the pathogenesis of HD in both mouse and cell studies, could be identified as a likely modifier of disease progression in HD [22], [26], [27]. The latest HD genetic modifier GWAS with more than 9,000 HD patients found an association with a *cis*-eQTL for increased *MSH3* expression in blood cells and identified additional loci with candidate modifier genes involved in DNA maintenance processes, *PMS1* (post-meiotic segregation increased 1 homolog), *PMS2* (post-meiotic segregation increased 2 homolog) and *LIG1* (DNA Ligase 1) [23].

Transcriptome-wide association studies (TWAS) integrating gene expression with GWAS data provided additional support for the role of DNA repair in disease onset. Here, the genes *FAN1*, *PMS1*, *PMS2* and *ASNSD1* were associated with later onset and increased expression and *MSH3* with decreased expression [23], [28].

## CAA-loss and CAACAG-duplication alleles as *cis*-acting AO modifiers

**Figure 1:**
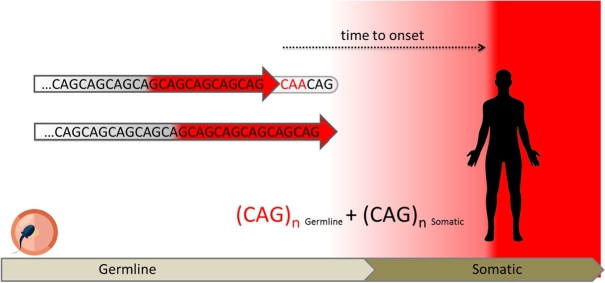
Interplay of inherited and somatic expansions to the progression of HD. The length of the *HTT* CAG repeat determines the disease phenotype. The disease occurs in individuals who have inherited a repeat tract that has expanded beyond a certain length. Individuals who carry an expanded HTT allele can become symptomatic at any time point in their life and be healthy until then with no apparent signs of the disease. The normal range of CAG repeats is present in unaffected individuals (CAG on a gray background). *HTT* alleles expanded into the pathological range (CAG on a red background) lead invariably to the onset of HD, and longer alleles are usually associated with an earlier onset of the disease. Although people with reduced-penetrance (RP) alleles have the chance of being asymptomatic throughout lifetime, there are always cases that become symptomatic earlier than expected. Some of these HD patients carry a loss of the penultimate glutamine-encoding CAA codon (in red) in their *HTT* allele. They have identical polyglutamine tract lengths as subjects with the frequent CAA codon, but exhibit a longer uninterrupted CAG sequence (red arrow), thus indicating that the pure number of uninterrupted CAG repeats (red arrow) makes a more decisive contribution to AO of HD than the number of encoded polyglutamines. *Cis*- and *trans*-acting modifiers like the loss of the CAA repeat interruption or variations in DNA repair genes appear to significantly affect the rate at which somatic expansions occur. Under the assumption that the repeat undergoes somatic expansion throughout lifetime, brain cells being most susceptible to disease pathogenesis in particular, HD becomes manifest and the first symptoms appear when the repeat length (the sum of the inherited and somatic expansions, CAG_n germline_ + CAG_n somatic_) exceeds a disease-specific threshold that may determine the onset of overt toxicity.

Another consistently significant GWAS signal is on chromosome 4 near *HTT*. However, since neither *HTT* promoter SNPs nor significant *cis*-eQTL SNPs could explain the significant signal, the *HTT* CAG repeat sequence itself came into focus again. In fact, previously described sequence variations in the *HTT* repeat sequence appear to be associated with the significant signal on chromosome 4 [29], [30], [31]. Typically, the *HTT* allele involves a pure CAG repeat that is followed immediately downstream by an additional glutamine-encoding CAA-CAG sequence. Thus, the total number of consecutive glutamines encoded by this region, which is measured by the standard diagnostic *HTT* PCR fragment-based genotyping assay, is typically equal to the number of pure CAGs, plus two glutamines encoded by the CAA-CAG sequence. Individuals carrying a loss of the penultimate CAA codon (i. e., CA**A**-CAG to CA**G**-CAG), therefore have an identical polyglutamine tract length as subjects with the frequent CAA codon, but exhibit a longer uninterrupted CAG sequence. Thus, in carriers of the loss of repeat interruption (LOI) diagnostic testing causes an underestimation of the uninterrupted CAG sequence by two repeats since the polyglutamine repeat length is inferred from fragment sizes based on the assumption of the common interrupting sequence (Figure 1).

Interestingly, LOI carriers show a significantly earlier onset than expected by their CAG length, whereas also existing rare CAA-CAG sequence duplications were associated with a delayed motor onset [23], [32], [33]. Identification of these *cis*-acting modifiers, therefore, indicates that the pure number of uninterrupted CAG repeats is the most significant contributor to AO of HD and not encoded polyglutamines.

Remarkably, the LOI variant was mainly observed in carriers of alleles with RP (36–39 CAGs), suggesting that the uninterrupted variant partially explains why some individuals who carry RP alleles manifest HD as early as mid-life, while others remain asymptomatic through advanced ages [34].

Thus, the *cis*-acting AO modifiers have implications for genetic diagnosis and counseling, especially when dealing with an intermediate or RP allele range. The underestimation by two CAG repeats in LOI carriers can have a profound effect on a possible disease manifestation. Therefore, diagnostic tests which currently do not assess the loss of the CAA codon should be adjusted, especially when dealing with individuals that carry alleles in the lower end of the disease-associated CAG range or when including patients in clinical studies with CAG repeat length inclusion criteria.

## But what underlies the phenotypic effects of the LOI variant?

Originally, the instability of tandem repeats was explained with the simple DNA slippage model, which postulates that repeats may be lost or gained during local misalignment of DNA during replication [35]. However, this model leaves some questions unanswered, including why repeat interruptions lead to the stabilization of repeat expansions.

Findings that interruptions of the CAG repeat within protein-coding transgenes mitigate toxicity and that an untranslated CAG repeat RNA can cause toxicity on its own further support a role of RNA in polyglutamine diseases. In a *Drosophila* model of SCA3, altering the CAG repeat sequence to an interrupted CAA-CAG repeat within the polyglutamine-encoding region did not affect ataxin-3 protein accumulation, but dramatically mitigated toxicity and the expression of an untranslated CAG repeat of pathogenic length conferred neuronal degeneration [36]. Naturally occurring interruptions found in several other trinucleotide repeat disorders like SCAs, myotonic dystrophy type 1 (DM1), Friedreich ataxia (FRDA) and Fragile X syndrome (FXS) have all been associated with the stabilization of the respective trinucleotide repeat loci and in some cases even with lower somatic instability and later AO [37], [38], [39], [40], [41], [42], [43], [44], [45], [46], [47], [48].

As early as 30 years ago, it was postulated that the ability of longer repeat regions to form stable secondary structures could lead to site-specific instability if the structure inhibits the action of replication or repair proteins at the repeat site [49]. It has been shown that trinucleotide repeat instability is mediated by the formation of unusual secondary structures that differ from the canonical B-DNA double helix during DNA replication, repair, recombination and gene transcription [50]. CAG repeats of sufficient length form imperfect stem and loop structures in transcripts [51]. HTT CAGs have been shown to form hairpins *in vitro* in a tripartite way, where the base is composed of interacting CAG and adjacent CCG repeats, followed by a central motif consisting solely of CAG repeats and a terminal section composed of the fold-back structure from CAG repeats [52]. The folding properties, however, depend on the interplay between repeated and specific flanking sequences, which have a significant influence on the correlation between the repeat length, the stability of the hairpin structure and the ability of this structure to trigger RNA pathogenesis in cells, suggesting a relevant role for the RNA structure towards inducing a toxic effect [51]. Different secondary structures may interfere with the progress of RNA polymerase and involve various DNA repair processes that can impact repeat expansion and contraction. The presence of repeat interruptions in such structures may facilitate their correct repair upon recognizing the mismatches that arise from the misalignment of repeat interruptions [53].

## Somatic instability

The *HTT* CAG repeat is unstable in germline and somatic cells, and expansion in both cell types has deleterious consequences. Germ line expansions are responsible for the intergenerational instability of the *HTT* CAG repeat, a phenomenon called anticipation. Transmission of the mutation to offspring is characterized by the tendency to expansion and the longer the repeat, the more severe the disease and the earlier the onset of symptoms. Already more than 25 years ago a highly significant correlation between CAG instability and the size of the parental CAG repeat as well as somatic mosaicism in sperm was described for paternal transmission in HD [54]. Later investigations in male transgenic mice showed that in germ cells, expansion is limited to the post-meiotic haploid cell, implying DNA repair mechanisms rather than DNA replication in DNA synthesis as the cause of the expansions [55]. Yet, somatic CAG repeat expansion also occurs in several other tissues, including the brain. Studies both in HD patient tissues and in HD mouse models have shown that the expanded *HTT* CAG repeat has a high tendency to further expand in somatic cells, whereas the degree of somatic instability is highly tissue-specific, repeat length-dependent and age-dependent [27], [56], [57], [58], [59], [60], [61], [62], [63], [64], [65], [66], [67], [68], [69], [70]. In transgenic and knock-in mouse models of HD, increased somatic CAG expansion is described in cells from the striatum, cortex and liver, whereas in cells from the cerebellum, blood and tail the repeat length is relatively stable [57], [63], [66], [71]. Large CAG repeat expansions with a similar distribution pattern like in mice were also demonstrated in post-mortem human tissues [60], [62], [64]. The latest study revealed a similar profile of tissue instability in seven adult and one juvenile HD patient that was also apparent in an individual with SCA1. Somatic CAG expansion was observed in all tissues, but to different degrees, with multiple cortical regions and neostriatum tending to have the greatest instability in the CNS and liver in the periphery [72]. These similar tissue-specific patterns of repeat expansion support the assumption that disease locus-independent *trans* factors cause the extent of CAG expansion in different cell types. Beside the inherited CAG length also previously identified DNA repair pathway-related modifier variations were shown to mediate somatic expansions in mouse models and humans. This supports the assumption that abnormal structure-dependent interactions of expanded RNA repeats with various cellular proteins and differing expression levels of repair genes might be the main or a contributing factor of the cell-specific vulnerability/CAG expansion [25], [26], [27], [32], [67], [73], [74].

## Conclusion

For nearly 30 years, scientists have been trying to uncover the basic principles that underlie the mechanisms of repeat instability. Especially in polyglutamine diseases the progressive, late-onset characteristics were initially attributed to a low accumulating toxicity of the polyglutamine proteins expressed from expanded CAG repeats. However, a synopsis of earlier and more recent studies shows that in addition to the abnormal function of the mutated protein also direct RNA-damaging effects or RNA-dependent mechanisms could represent a mechanistic basis for the molecular pathogenesis of HD.

For HD as well as several types of SCA, variation in DNA repair proteins as well as sequence interruptions have been identified as major modifiers of AO and disease progression, implicating ongoing somatic expansions as a common mechanism of disease. Thus, there is much to suggest that the combination of the number of inherited repeats together with the degree of somatic instability determines the AO for a given disease and patient (Figure 1).

There is currently no cure for HD and pharmacotherapy is limited to symptomatic treatment of movement disorders and psychiatric symptoms. For the moment, the most promising treatment appears to be targeting the pathological processing of *HTT* mRNA, or upstream. A number of RNA-targeting therapies have recently entered clinical trials, which aim to lower the mutant HTT production by the use of antisense oligonucleotides (ASOs) and RNAi. However, each of the different strategies has specific advantages and disadvantages, as recently seen by the failures of Roche and Wave Therapeutics’s ASO candidates in HD [75].

Considering the influence of somatic instability on disease onset and progression, targeting the repeat instability is a very attractive disease-modifying strategy in HD and related expansion disorders, and possibly suppressing or reversing somatic repeat expansion may halt or delay the progression of the disease.
